# Re: “Chronic high consumption of energy drinks and cardiovascular risk in adolescents - results ofthe EDKAR study”

**DOI:** 10.1007/s10654-025-01339-1

**Published:** 2026-02-21

**Authors:** Nikolaus Alexander Haas, Delphina Gomes, Maria Jaros, Robert Dalla Pozza, Meike Schrader, Felix Sebastian Oberhoffer

**Affiliations:** Department of Pediatric Cardiology and Intensive Care, Ludwig Maximillians University, Marchioninistr. 15, Campus Grosshadern, Munich, Germany

With great interest we read the article “Chronic high consumption of energy drinks and cardiovascular risk in adolescents—results of the EDKAR-study” by Menzel et al. published in your distinguished journal [1]. This article carries significant and important data regarding the topic of Energy Drink (ED) usage in teenagers and young adults and the potential effects on cardiovascular health, we would however like to raise the following points for discussion:

## Study protocol

The inclusion and exclusion criteria are not clearly defined and the exact circumstances of investigations (i.e. with or without energy drink consumption, see below) are not defined. Both groups were not matched in terms of baseline characteristics. Both groups differed significantly in multiple baseline characteristics. Compared to the control group, chronic high ED consumers were older, had a higher body mass index, and reported significantly greater use of tobacco, alcohol, and marijuana. Additionally, there were differences between both groups in terms sleeping pattern and of educational background.

## Cardiovascular measurements

In addition, it remains unclear at what time during the day these examinations were conducted, when energy drinks (ED) were consumed in relation to the cardiovascular examinations, and after what time span of ED drinking pause these investigations (BP, ECG, ECHO, etc.) were performed. As an ED drinking pause for the morning or day of investigation seems likely for teenagers visiting this assessment at the university hospital, the parameters obtained may well represent the trough values and not the real-life status of high ED consumers. These factors may have introduced significant confounding effects on the cardiovascular outcome measures.

We believe that the interpretation of a conventional 12-lead ECG is insufficient for assessing the risk for arrhythmia; a 24-hr Holter ECG seems obligatory for a more accurate evaluation and further discussions. Similarly, 24-hour blood pressure monitoring is needed for reliable conclusions regarding arterial hypertension. To detect subtle vascular changes in this young population, endothelial function, arterial stiffness and carotid intima-media thickness should be assessed. Cardiometabolic risk factors (e.g. prevalence of overweight, sugar metabolism disorders) were not addressed. These results presented can therefore not be used for a general cardiac risk assessment of ED consumption.

## Data adjustment to pediatric reference values

This study included adolescent participants. However, adult reference values adult refernce values only were used to interpret cardiovascular outcome parameters. We consider this approach as inappropriate, as dedicated pediatric reference values exist specific to age, sex, body height, and body weight. Therefore these results must be presented and discussed as Z-score values accordingly. For better assessment of the results reported, absolute body weight and height parameters are lacking in Table 1. In addition to all anthropometric parameters, this implies also for all ECHO parameters (i.e. septum thickness, LVEDD, Global longitudinal strain, MAPSE, TAPSE) or ECG parameters.

## Data interpretation

As acute ED consumption increases blood pressure, chronic ED consumption may result in chronic higher blood pressure and secondary cardiac reactions. The diastolic blood pressure increased substantially from a mean of 74 mmHg in the control group (*n* = 160), to a mean of 75,6 in the group of high consumers (*n* = 97) and to 79 mmHg in those defined as chronic high consumers. Similarly, the ECHO data showed an increase of the septum thickness from 8 mm to 8,7 and 9 mm, the E wave declined from 90 cm/sec to 85 cm/sec and 81,5 cm/sec, the A-wave from 50 cm/sec to 50 cm/sec and 43 cm/sec accordingly, all indicators of an impairment in the diastolic function. So, these subtle trends are reported even in the young subjects after a short time of ED usage. These findings may indicate indirect signs of chronically elevated left ventricular afterload, possibly due to continuous or episodic increases in arterial blood pressure, within this cohort and may become even more relevant and apparent after several years of chronic ED consumption.

## Definition of doses and inclusion criteria

As cited by the authors, the European Food Safety Authority (EFSA) derived safe caffeine quantities for healthy people in 2015 [2] and high ED consumers are defined accordingly [3]. According to EFSA, individual caffeine doses of up to three mg caffeine per kilogram (kg) body weight, i.e. roughly 200 mg (standardized 75 kg person) are still of no health concern for adults and chronic use. For habitual consumption, up to 5.7 mg per kg body weight, or around 400 mg, are regarded as of no concern for healthy adults. The available literature suggests that cardiovascular effects experienced by adult caffeine consumers at levels up to 600 mg/day (or about 8.5 mg(kgbw) are in most cases mild, transient, and reversible, with no lasting adverse effect [4, 5]. In the Table 1 presented, the absolute ED doses, the body weight, the relative doses of caffein etc. are unfortunately not presented. This seems however crucial in order to estimate the doses consumed - a presentation only in the appendix seems unfavorable. In Table 8 of the appendix, the absolute caffein doses were reported. Based on an average estimated weight of 70–80 kg of an 18-year-old, the relative doses of 5.24 mg/kg/day and 8.63 mg/kg/day translate to about 400 mg daily dose and 600 mg daily dose. Again, it is not clear in the setting of this investigation, if the teenagers investigated did have any energy drink consumption on the day of investigation. Based on the results presented and considering that young individuals with a relatively short high chronic ED consumption behavior (≥ 12 months) were included, we find these findings worrisome and that the 400 and 600 mg doses recommendations should be excluded for adolescents.The conclusion of the authors that these dosages may be not harmful, is not supportedby these data.

## Loss-to-follow-up

We raise our concerns to the statement: “Nevertheless, the present study achieved satisfactory response rates in both groups (chronic high ED consumption: 34.4%, control group: 37.7%)”. This may well be regarded as a relatively large loss-to-follow-up and imply a non-response bias; it is possible that adolescents who chose to participate differed systematically from those who did not.

## Conclusion

In summary we congratulate the authors for presenting all the concomitant lifestyle factors associated with the habits of the high ED consumers. We however strongly disagree that the setting of the EDKAR study and the data presented may give reliable information on the cardiovascular risk of chronic ED consumption at higher levels.
